# Intraoperative zero-heat-flux thermometry overestimates esophageal temperature by 0.26 °C: an observational study in 100 infants and young children

**DOI:** 10.1007/s10877-020-00609-5

**Published:** 2020-10-31

**Authors:** Marcus Nemeth, Marijana Lovric, Thomas Asendorf, Anselm Bräuer, Clemens Miller

**Affiliations:** 1 Department of Anesthesiology, University Medical Centre Göttingen, Robert-Koch-Str. 40, 37075 Göttingen, Germany; 2 Department of Medical Statistics, University Medical Centre Göttingen, Göttingen, Germany

**Keywords:** Body temperature, Zero-heat-flux, Pediatric, Child, Anesthesia, Monitoring

## Abstract

In pediatric anesthesia, deviations from normothermia can lead to many complications, with infants and young children at the highest risk. A measurement method for core temperature must be clinically accurate, precise and should be minimally invasive. Zero-heat-flux (ZHF) temperature measurements have been evaluated in several studies in adults. We assessed the agreement between the 3M Bair Hugger^™^ temperature measurement sensor (T_ZHF_) and esophageal temperature (T_Eso_) in children up to and including 6 years undergoing surgery with general anesthesia. Data were recorded in 5 min-intervals. We investigated the accuracy of the ZHF sensor overall and in subgroups of different age, ASA classification, and temperature ranges by Bland–Altman comparisons of differences with multiple measurements. Change over time was assessed by a linear mixed model regression. Data were collected in 100 children with a median (1st–3rd quartile) age of 1.7 (1–3.9) years resulting in 1254 data pairs. Compared to T_Eso_ (range from 35.3 to 39.3 °C; median 37.2 °C), T_ZHF_ resulted in a mean bias of +0.26 °C (95% confidence interval +0.22 to +0.29 °C; 95% limits of agreement −0.11 to +0.62 °C). Lin’s concordance correlation coefficient was 0.89. There was no significant or relevant change of temperature over time (0.006 °C per hour measurement interval, p = 0.199) and no relevant differences in the subgroups. Due to the mean bias of +0.26 °C in T_ZHF_, the risk of hypothermia may be underestimated, while the risk of hyperthermia may be overestimated. Nevertheless, because of its high precision, we consider ZHF valuable for intraoperative temperature monitoring in children and infants.

**Electronic supplementary material:**

The online version of this article (10.1007/s10877-020-00609-5) contains supplementary material, which is available to authorized users.

## Introduction

Perioperative normothermia is an important quality metric in pediatric anesthesia [1, 2]. Despite significant efforts [3], perioperative hypothermia is still common in children and has to be considered a severe complication leading to acidosis, coagulopathy, or apnea [4]. Neonates, infants, and young children are at increased risk of perioperative hypothermia due to their reduced weight-to-surface-area-ratio and limited subcutaneous fat stores [5]. Particularly in this age group, however, there is also a clear risk of overheating, which is associated with relevant complications such as surgical site infections [6]. Temperature management requires an accurate method to measure core temperature.

In children, intraoperative core temperature is usually measured in the nasopharynx, esophagus, bladder, or rectum. Esophageal temperature measurement most accurately reflects blood temperature and is an overall accepted surrogate measure of core temperature [7]. Although all these methods are less invasive than pulmonary artery catheter placement, they can still harm the children’s vulnerable mucosa. Therefore, an accurate, but less or even non-invasive, quick to apply monitor would be preferable, which could ideally be used in the entire perioperative period.

Introduced in 2014, the 3M Bair Hugger^™^ Temperature Monitoring System (3M USA, St. Paul, MN), formerly known as SpotOn™ [8], is a non-invasive cutaneous temperature sensor based on zero-heat-flux technology (ZHF). It consists of two thermometers, separated by an insulator and a covering servo-controlled heater [9]. An isothermal tunnel corrects the varying temperature of the skin surface [9]. Multiple studies confirmed its clinically acceptable accuracy in adults [9–15], but there is limited evidence in children [16, 17]. A recent meta-analysis revealed substantial differences and a need for further studies in children was formulated [8].

We assessed the level of agreement between ZHF temperature measurements (T_ZHF_) and distal esophageal temperature (T_Eso_) in children up to and including 6 years of age.

## Methods

Institutional Review Board approval for this prospective single-center observational study was granted by the local ethics committee (Ethics committee of the University Medical Centre Göttingen, No. 25/1/19). Written informed consent was obtained from parents or legal guardians before enrollment. The study was registered in the German Clinical Trials Register (DRKS-ID: DRKS00016655) on 03/26/2019 before enrollment of the first patient. We followed STROBE guidelines for reporting of observational studies [18].

*Inclusion criteria* were children up to and including 6 years undergoing surgery with general anesthesia and a minimal scheduled operation time of 30 min. Airway management was performed with a 2nd generation laryngeal mask or endotracheal intubation.

*Exclusion criteria* were refusal to participate, premature birth <37 weeks of gestation, cardiothoracic operations, and oral operations in which the surgical field would have impeded the positioning of the esophageal probe (e.g. ENT).

### Study protocol

Patients were placed on the already warmed pediatric underbody blanket (Moeck Warming System^™^, Hamburg, Germany), which was continued for the whole session using a forced-air warming system. The ambient temperature of the pediatric operating room had a constant temperature level of approximately 24 °C as recommended [19]. Immediately after induction of general anesthesia, the temperature sensor of the 3M Bair Hugger^™^ Temperature Monitoring System Model 370 was placed on the patient’s lateral forehead. After securing the airway, an esophageal temperature probe (RÜSCH Temperature Sensor^™^, Teleflex Medical, Athlone, Ireland) was placed either through the drainage canal of the laryngeal mask airway (AmbuAuraGain^™^, Ballerup, Denmark) or orally when an endotracheal tube was used. Insertion depth was calculated for each patient according to the formula published by Whitby und Dunkin aiming to place the tip of the probe in the distal fourth of the esophagus [20].

Both devices were connected to the monitoring system (PhilipsIntelliVue MX700^™^, Hamburg, Germany) and pairs of temperature values were recorded in five-minute intervals. Recordings started after the ZHF sensor finished calibration of approximately 3 min [16]. Both, temperature probe and sensor, were removed before the emergence of anesthesia.

The following parameters were documented: biometric data (age, weight, height, sex), indication for surgery, operative procedures, duration of measurement and the occurrence of any reactions or lesions to the skin of the forehead.

### Data analysis

We analyzed the occurrence of clinically relevant differences regarding the measurement accuracy of the ZHF sensor in patients overall, over time and in subgroups of different age, ASA classification and temperature ranges. Hypothermia was defined as T_Eso _<36.0 °C and hyperthermia was defined as T_Eso_ >38.0 °C [21].

### Statistical analysis

For our primary hypothesis we used a Bland–Altman comparison of differences with multiple measurements [22]. A sample size of 100 patients with an even number of measurements per patient was considered sufficient to demonstrate a clinically meaningful difference, as there are no formal rules for power calculations for this method. Further, we calculated the proportion of all differences that were within a predefined threshold of ±0.50 °C of T_Eso_ [23] and Lin’s concordance correlation coefficient to assess the agreement between pairs of observations [24].

The change in temperature difference between methods over time was assessed by univariate linear mixed model regression. Subgroups were analyzed by multivariate linear mixed models. Regarding subgroups, group differences were assessed by type III analysis of variance (ANOVA) with Kenward–Roger’s approximation [25]. Accuracy (bias reflecting mean differences between methods) and precision (limits of agreement within ±1.96 standard deviations) were calculated using estimated marginal means [26] and compared to the full population. Data were analyzed using Excel (Microsoft^®^ Excel^®^ for Microsoft 365, Redmond, WA, USA), MedCalcVersion 19.2.1 (MedCalc Software Ltd, Ostend, Belgium), and R 3.6.2 (R Core Team, 2019, Vienna, Austria).

## Results

**Fig. 1:**
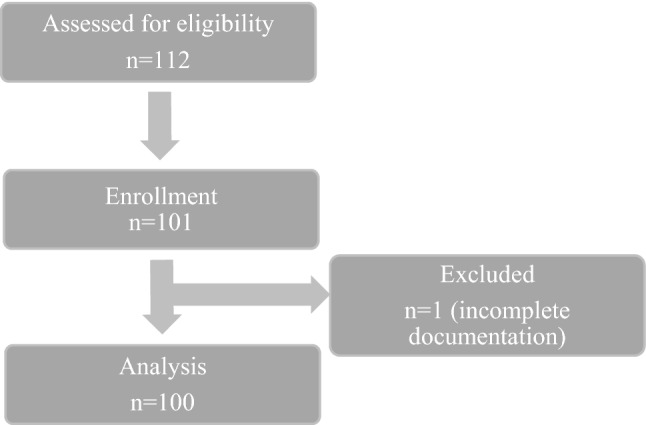
Flow diagram of enrollment

We enrolled 101 subsequent children (of which 23 female) 0–7 years of age with a median (1st–3rd quartile) age of 1.7 (1–3.9) undergoing surgery with general anesthesia between April and September 2019. One patient was excluded due to incomplete documentation (see Fig. 1). Median measurement duration of 40 min (1st–3rd quartile: 25–75 min) per patient allowed an analysis of 1254 data pairs. The participants’ characteristics are presented in Table 1.

**Table 1:** Participant characteristics

	Number of patients or mean (1st–3rd quartile)	Number of records
Overall	100 (23 female)	1254
Weight (kg)	12.0 (10.0–16.0)	
BMI (kg/m²)	15.6 (14.6–17.8)	
Age (years)		
<1	23	361 (28.8%)
≥1 and<2	32	379 (30.2%)
≥2 and <3	6	93 (7.4%)
≥3 and <4	14	147 (11.7%)
≥4 and <5	9	78 (6.2%)
≥5 and <6	10	124 (9.9%)
≥6 and <7	6	72 (5.7%)
ASA physical status		
I	60	542 (43.2%)
II	19	405 (32.3%)
III	19	262 (20.9%)
IV	2	45 (3.6%)
Type of surgery		
Urological	59^*^	639 (51.0%)^*^
Visceral	31^*^	345 (27.5%)^*^
Trauma and orthopedic	11	251 (20.0%)
Neurosurgical	5	93 (7.4%)

^*^Six patients had urological and visceral surgery at the same time

**Fig. 2:**
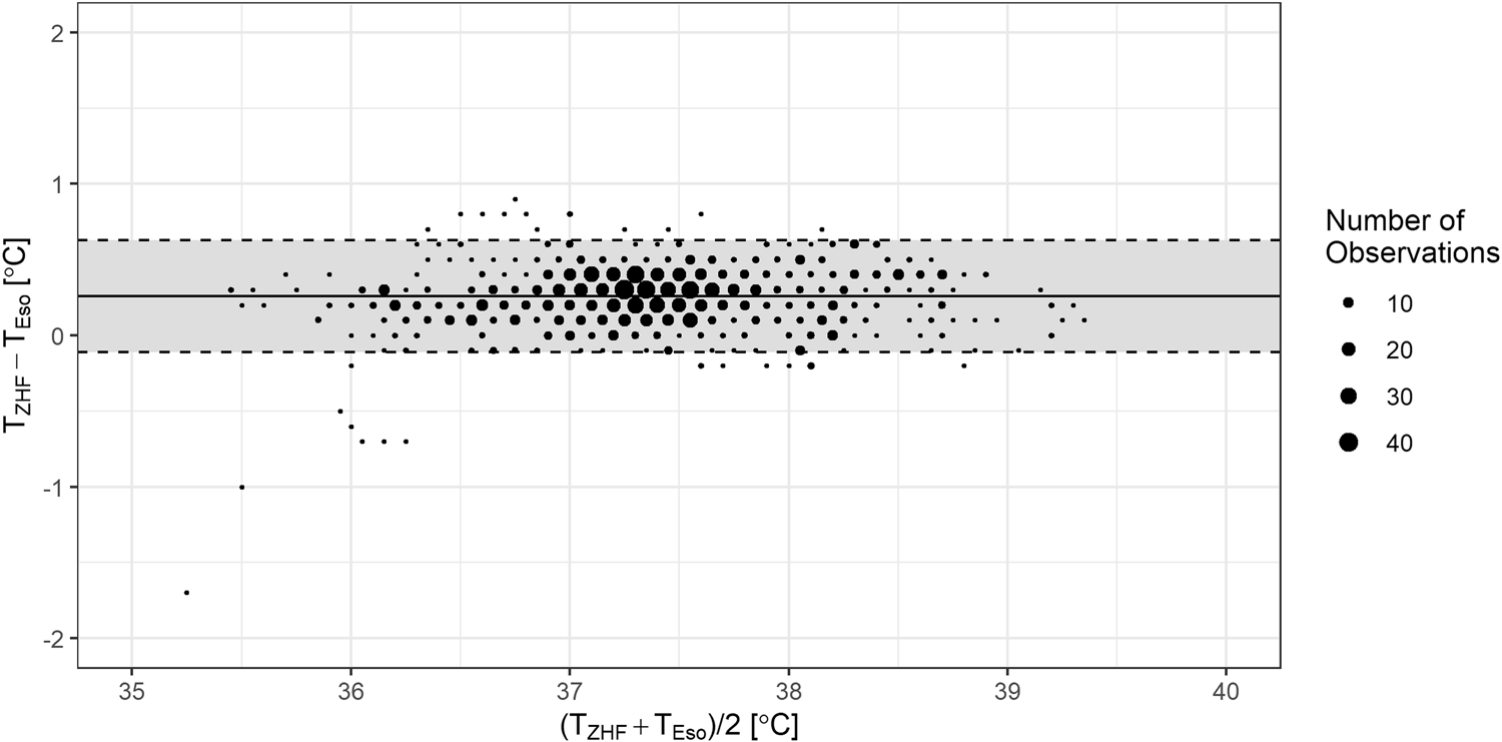
Bland–Altman plot with multiple temperature measurements (100 patients with 1254 measurement pairs) of the ZHF sensor (T_ZHF_) and an esophageal probe (T_Eso_). Solid line indicates mean bias (+0.26 °C) and dashed lines 95% limits of agreement (LOA). Upper LOA: +0.62 °C, lower LOA: −0.11 °C

The measurements of T_Eso_ ranged from 35.3 to 39.3 °C with a median (1st–3rd quartile) of 37.2 °C (36.9–37.6 °C). T_ZHF_ measurements ranged from 34.4 to 39.4 °C with a median (1st–3rd quartile) of 37.5 °C (37.2–37.8 °C). Compared to T_Eso_, T_ZHF_ measurements resulted in a mean bias of +0.26 °C with 95% limits of agreement within −0.11 to +0.62 °C (see Fig. 2). 95.7% of measured temperature differences where within ±0.50 °C of T_Eso_. Lin’s concordance correlation coefficient was 0.89 (95% CI: 0.88–0.90).

**Fig. 3:**
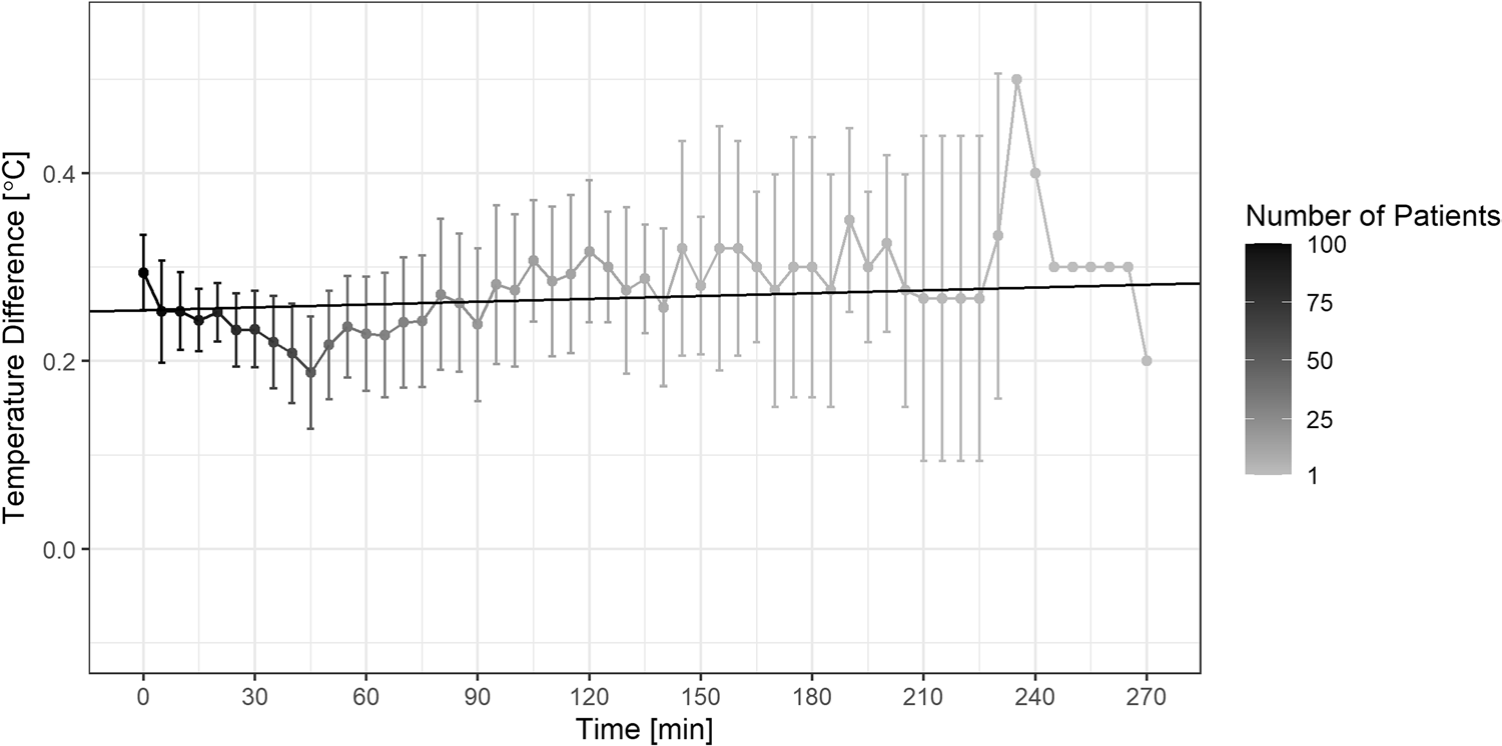
Regression line of temperature measurements over time. The regression line shows average increase of temperature difference over time of 0.006 °C per hour measurement interval (p = 0.199; intercept = 0.26 °C). Please note, number of patients decreased over time due to different durations of surgery

Differences in temperature between T_ZHF_ and T_Eso_ did not change significantly over time (0.006 °C per hour measurement interval, p = 0.199, Fig. 3). Raw data for each measurement method are shown as supplementary material.

**Fig. 4:**
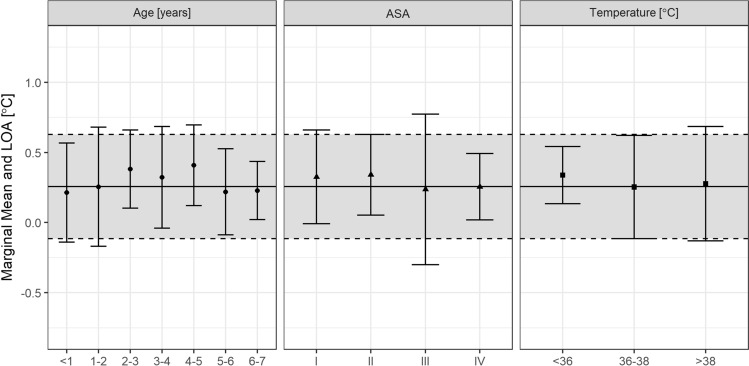
Subgroups of age, ASA classification (American Society of Anesthesiologists physical status) and temperature range of esophageal probe with mean bias reflected by circle, rectangle, or triangle, respectively, and whiskers indicating the 95% limits of agreement of the subgroup. Solid line indicates mean bias and dashed lines 95% limits of agreement of overall measurements

Subgroup analysis (see Fig. 4) revealed statistically significant measurement differences between temperature range of esophageal probe (p = 0.0054), but no statistical significance of age, or ASA classification (see Table 2).

**Table 2:** Results of the type III mixed effects ANOVA with Kenward–Roger’s approximation of degrees of freedom and difference of temperature measurements as dependent variable

	Sum of squares	Degrees of freedom (numerator; denominator)	F-statistic	p-value
Time	0.006415	1; 1170	0.674	0.4118
Age group	0.1189	1; 90.02	2.081	0.0631
ASA classification	0.04264	3; 87.83	1.493	0.2219
Temperature range of esophageal probe	0.09957	2; 1195	5.231	0.0054

Interaction terms are not included

Hypothermia occurred in 3 patients during surgery (2 patients at the end of measurement). In 2 of these, the ZHF sensor indicated normothermia. In contrast, 14 patients became hyperthermic during surgery, while the ZHF sensor indicated temperatures above 38 °C in 27 patients.

The ZHF sensor was well tolerated in all patients and no burn or skin reaction was observed during the study period.

## Discussion

The 3M Bair Hugger^™^ Temperature Monitoring System showed a mean bias of +0.26 °C (95%-CI +0.22 °C to +0.29 °C) when compared against a widely-used standard of temperature measurement in the distal esophagus in 100 infants and young children. Differences of systemic overestimation in subgroups of ASA classification, age, or temperature range varied only minimally and did not change significantly over time.

When evaluating new measurement methods, identifying the gold standard is crucial. Many studies in adults defined blood temperature in the pulmonary [9, 11] or iliac [12] artery as the most suitable reference method. However, blood temperature can be affected by organ replacement therapy or cold fluid infusion, which is probably the main source of error in the perioperative setting. Although blood temperature can be feasible as a reference method in adults, it is not suitable for children undergoing non-cardiac surgery [9, 11, 12]. Thus being less invasive, the esophageal probe is the most appropriate method [7, 16, 23]. Correctly placed, it lies directly between the left atrium and the descendent aorta and is therefore far away from the potentially cooling airway [20].

Our main finding of a positive bias is in accordance with a previously published study in children by Carvalho et al. [16] who found a bias of +0.14 °C with 95% limits of agreement of −0.39 to 0.66 °C. Although our observed bias of +0.26 °C was relatively high, the limits of agreement were tight. Concerning these results together with a high rate of all T_ZHF_ measurements being within the predefined threshold of T_Eso_, one might conclude a clinically acceptable accuracy of the two measurement methods. This is emphasized by a higher Lin´s correlation coefficient than in previous studies [15, 16].

The mean bias varied only minimally in the subgroups of age, ASA classification, temperature range, and in the duration of measurement. T_ZHF_ worked adequately within the tested subgroups. Although statistics showed a significance of temperature range between T_Eso_ < 36 °C to T_Eso_ > 38 °C, the mean bias varied only by 0.1 °C. We do not consider this small difference clinically relevant. However, a single patient undergoing neurosurgery (implantation of a shunt between an arachnoid cyst and the peritoneum), caused a larger deviation in some phases of the measurement. This may have been caused by the proximity of the sensor to the surgical diathermy. With the discrepancy of incidences in hypothermia and hyperthermia, our study remarkably underlines the risk of children becoming hyperthermic due to highly effective forced-air warming devices with the potential of overheating [3]. This can also lead to harm such as surgical site infection, thermal discomfort, or sweating in the postanesthetic care unit [6].

There are some interesting aspects of our study compared to Carvalho et al. [16]. In addition to nearly twice as many children being enrolled, we also included a high number of infants. Thus, our data support the accuracy of T_ZHF_ in infants. Further, in contrast to the study of Carvalho et al. [16] there was no noticeable temperature drop after the induction of anesthesia, probably because all our patients received active warming therapy already prior to the induction of anesthesia.

The only other study in pediatric anesthesia also revealed a higher bias of T_ZHF_ [16] in contrast to biases below zero in adults [9, 11–13, 27]. There are several possible causes for these differences. First, in children intraoperative warming systems might be much closer to the head compared to adults leading to a quicker warming up [19]. Second, heads of infants and children consist of thinner skull bones resulting in a closer proximity of the skin to the highly perfused brain [28]. Additionally, if the device uses correction algorithms that are based on the anatomical features of an adult, slight overestimation of temperature in young children might be the consequence that Carvalho et al. [16] and we have observed.

The question why T_ZHF_ slightly overestimates temperature in infants and children may be interesting from a technical point of view. However, from a clinical point of view, a more relevant question is whether this difference is acceptable for clinical practice in pediatric anesthesia? Many studies comparing new temperature monitoring devices to a gold standard defined a combined inaccuracy (bias and limits of agreement) smaller than 0.5 °C to be accurate enough [9, 29, 30]. In our opinion, this requirement is very high and most studies investigating new non-invasive thermometers could not determine an accuracy meeting this criterion [9, 29, 30]. Still, all these studies concluded that the new devices were accurate enough for daily anesthetic practice [9, 16, 29, 30].

However, as consequence of the bias that we have observed, users should be aware that it might be possible that a child is already hypothermic when temperatures just around 36 °C are measured with a ZHF sensor.

### Limitations

This study has important limitations. First, like other studies, we applied T_Eso_ as a reference method [16] which may not be as accurate as directly measured blood temperature but has been proven to correlate well with core body temperature derived with pulmonary artery temperature measurements [7, 23]. Second, by using equidistant 5-min-intervals we may not have detected an eventual time lag within these intervals, especially compared to other studies using shorter time-intervals [15, 16]. However, a 5-min-interval was shown to be efficient [15]. Third, the used age-based formula for insertion depth of the esophageal temperature probe in children slightly differs from other recently published recommendations based on height [31]. Fourth, we cannot make any statement about the validity in the presence of severe hypothermia as we only included elective surgical patients with the goal of maintaining normothermia. Investigations of ZHF in extremer temperature ranges are required [16]. Furthermore, the ambient temperature of the pediatric operating room was not monitored separately for study purposes. Although it has a constant temperature level, the non-standardization may be closer to the clinical reality. This also applies to the wide inclusion criteria chosen: it could increase the bias but is closer to the real-world conditions. Therefore it might provide a better generalizability of the results [16].

## Conclusion

Temperatures in infants and young children obtained with the 3M Bair Hugger^™^ temperature monitoring system showed a mean bias of +0.26 °C compared to an esophageal probe, regardless of the duration of measurement, age, ASA classification, and temperature range. The risk of perioperative hypothermia may be underestimated, while the risk of hyperthermia may be overestimated. Nevertheless, because of its high precision (95% limits of agreement −0.11 to +0.62 °C), we consider ZHF valuable for intraoperative temperature monitoring in children and infants.

## Electronic supplementary material

Below is the link to the electronic supplementary material.

Supplementary file1 (TIFF 8438 kb)

Supplementary file2 (TIFF 8438 kb)

## Data Availability

Department of Anesthesiology, University Medical Centre Göttingen, Germany. Not applicable.
