# Supplementary figures and images for: Intraoperative zero-heat-flux thermometry overestimates esophageal temperature by 0.26 °C: an observational study in 100 infants and young children
Source: J Clin Monit Comput. 2020 Oct 31;35(6):1445–51. doi: 10.1007/s10877-020-00609-5 (PMC8542556; doi:10.1007/s10877-020-00609-5)

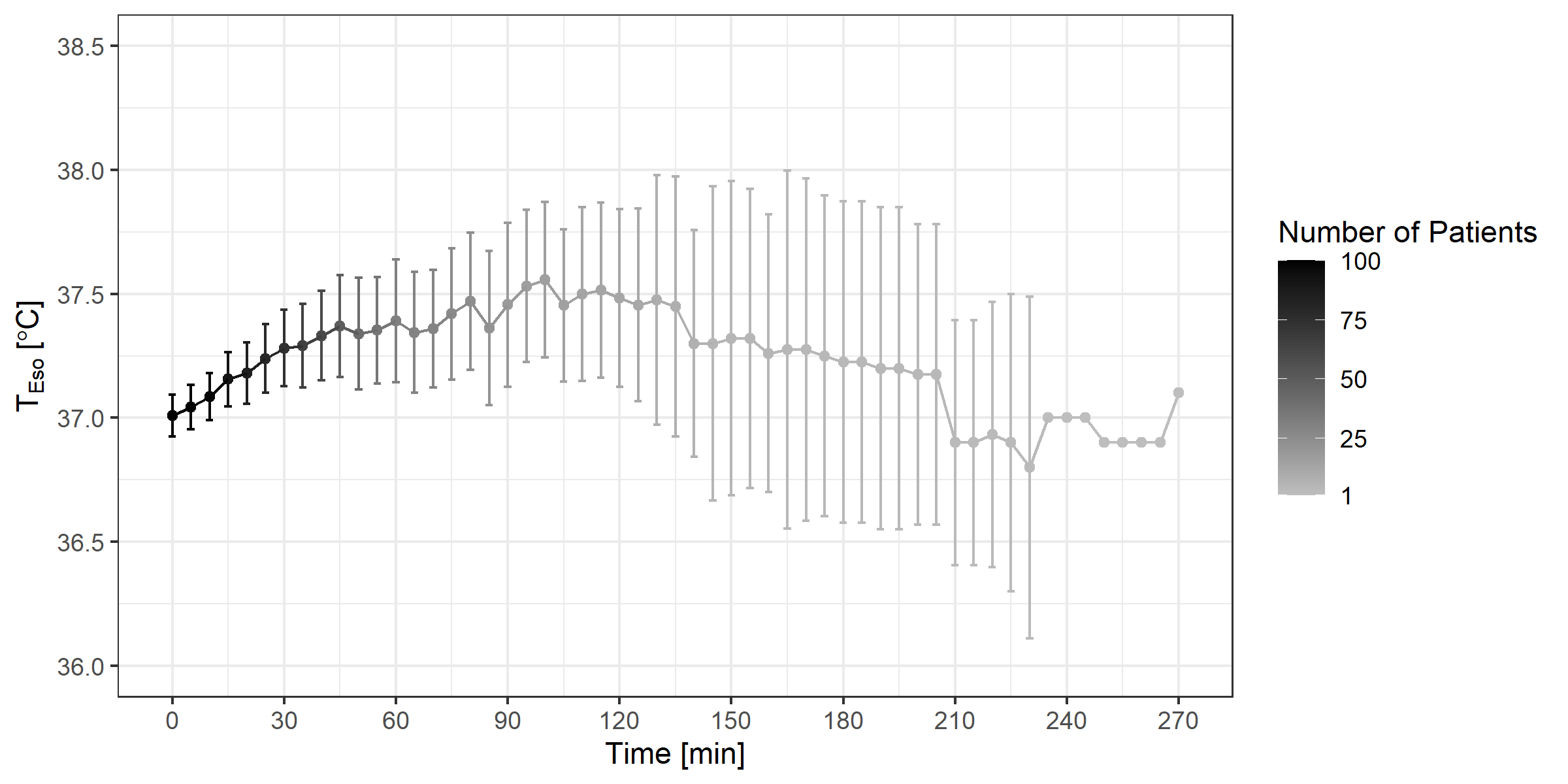

Supplement: Supplementary file 1 — Supplementary file1 (TIFF 8438 kb) [file 10877_2020_609_MOESM1_ESM.tiff]

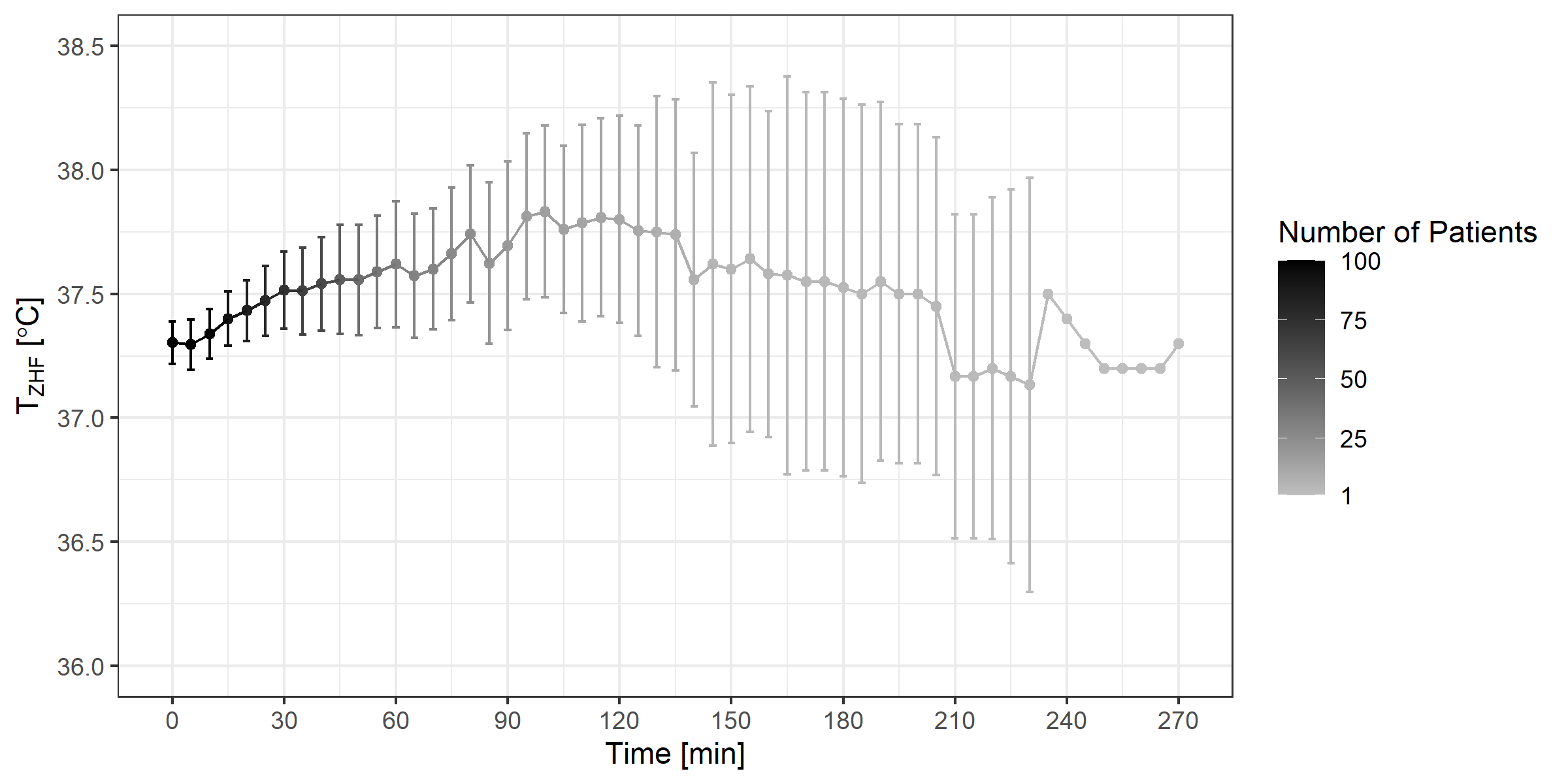

Supplement: Supplementary file 2 — Supplementary file2 (TIFF 8438 kb) [file 10877_2020_609_MOESM2_ESM.tiff]
